# The Impact of Age on Clinical Outcomes of Coronary Artery Bypass Grafting: Long-Term Results of a Real-World Registry

**DOI:** 10.1155/2017/9829487

**Published:** 2017-12-20

**Authors:** Francesco Nicolini, Daniela Fortuna, Giovanni Andrea Contini, Davide Pacini, Davide Gabbieri, Claudio Zussa, Rossana De Palma, Antonella Vezzani, Tiziano Gherli

**Affiliations:** ^1^Cardiac Surgery Unit, Department of Medicine and Surgery, University of Parma, Parma, Italy; ^2^Regional Agency for Health and Social Care, Emilia-Romagna, Italy; ^3^Cardiac Surgery Unit, Surgery Department, Parma Hospital, Parma, Italy; ^4^Cardio-Thoracic-Vascular Department, University Hospital S. Orsola-Malpighi, Bologna, Italy; ^5^Department of Clinical Cardiology and Thoraco Vascular Surgery, Hesperia Hospital, Modena, Italy; ^6^Department of Cardiology and Cardiac Surgery, Villa Maria Cecilia Hospital, Lugo, Ravenna, Italy

## Abstract

The aim of this retrospective multicenter registry study was to investigate age-dependent trends in mortality, long-term survival, and comorbidity over time in patients who underwent isolated CABG from 2003 to 2015. The percentage of patients < 60 years of age was 18.9%. Female sex, chronic pulmonary disease, extracardiac arteriopathy, and neurologic dysfunction disease were significantly less frequent in this younger population. The prevalence of BMI ≥ 30, previous myocardial infarction, preoperative severe depressed left ventricular ejection fraction, and history of previous PCI were significantly higher in this population. After PS matching, at 5 years, patients < 60 years of age reported significantly lower overall mortality (*p* < 0.0001), cardiac-related mortality (*p* < 0.0001), incidence of acute myocardial infarction (*p* = 0.01), and stroke rates (*p* < 0.0001). Patients < 60 years required repeated revascularization more frequently than older patients (*p* = 0.05). Patients < 60 who underwent CABG had a lower risk of adverse outcomes than older patients. Patients < 60 have a different clinical pattern of presentation of CAD in comparison with more elderly patients. These issues require focused attention in order to design and improve preventive strategies aiming to reduce the impact of specific cardiovascular risk factors for younger patients, such as diet, lifestyle, and weight control.

## 1. Introduction

Premature coronary artery disease in young patients (CAD) is a rapidly progressive form of the disease [1], but it requires invasive revascularization infrequently [2, 3].

Numerous studies reported that young patients with CAD have a significant prevalence of classic cardiovascular risk factors [4, 5], and that the premature clinical onset of their symptoms can be more aggressive than in elderly patients [6]. In fact, young adults who undergo coronary artery revascularization are a specific subpopulation of patients, and to date there have been few studies on survival data, cardiovascular events, or the need for repeated revascularization [3, 7].

It is clear that the results of revascularization in young patients need to be durable, in order to avoid recurrence of symptoms or cardiovascular events and the need for repeat revascularization. Young patients undergoing coronary artery bypass grafting (CABG) demonstrate survival rates similar to those of percutaneous coronary intervention (PCI), but lower rates of repeated revascularization [7]. The majority of recent studies of CABG outcomes investigate only the risks for elderly patients undergoing coronary revascularization [8–12], but there are few long-term reports of the impact of age stratification on CABG outcomes, particularly for young patients.

The aim of this retrospective multicenter registry study was to investigate age-dependent trends in mortality, long-term survival, and comorbidity over time, in a population of patients who underwent isolated CABG between 2003 and 2015.

## 2. Materials and Methods

### 2.1. Data Source

Emilia-Romagna (ER) is an Italian region with about 4 million inhabitants. Cardiac surgery is performed by six hospitals (two public university hospitals and four private hospitals). The RERIC Registry (Registro dell'Emilia Romagna degli Interventi Cardiochirurgici) is a prospective regional database designed in 2002 by the Agency for Health and Social Care of ER region, with the aim of collecting pre-, intra-, and postoperative data from all the patients undergoing cardiac surgical procedures in the region. The rationale and methodology of RERIC have been published previously [12, 13]. The Regional Agency for Health and Social Care ensures data quality/completeness control. The RERIC registry is linked to the ER regional mortality registry and to the regional hospital admission database, in order to collect accurate information on the occurrence of follow-up mortality and morbidity. Variables and events occurring after the index hospital discharge have also been collected from outpatient clinics at the individual Institutions. In case of absent/missing data, variables and events have been collected by direct phone contact with general practitioners and only if persistently missed by phone contact with patients and families. This registry is based on current clinical practice. The requirement for individual patient consent was waived because of the retrospective design of the study and because data were collected from routine care procedures. All data were anonymized and deidentified prior to analysis by the central statistical laboratory of the Regional Agency for Health and Social Care. The protocol of the study is in accordance with the Declaration of Helsinki.

### 2.2. Study Population

**Figure 1:**
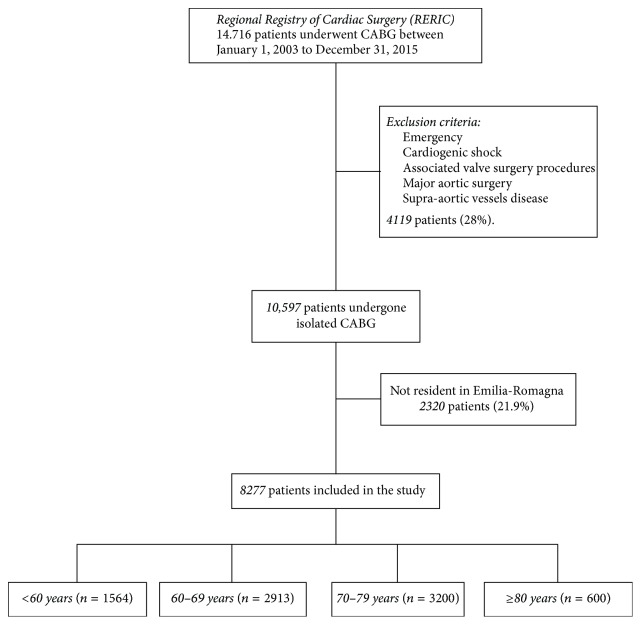
Selection criteria.

From January 1, 2003 to December 31, 2015, data of all patients undergoing CABG were collected in the RERIC Registry. Exclusion study criteria were emergency, cardiogenic shock, associated valve surgery procedures, major aortic surgery, and supra-aortic vessels disease requiring surgery. Patients with a previous cardiac operation and requiring only isolated CABG at the time of RE-DO surgery were included in the study. After these exclusions, we filtered 10,597 patients subjected to isolated CABG. Additional exclusion criteria were not being resident in ER (administrative follow-up not feasible for 2320 patients) and the presence of incomplete information about baseline and procedural characteristics. The remaining 8277 patients were followed through January 30, 2017. The patients were divided into four age bands for the purposes of the statistical analysis: <60 years (*n* = 1564), 60–69 years (*n* = 2913), 70–79 years (*n* = 3200), and ≥80 years (*n* = 600) (Figure 1).

### 2.3. Procedures

Decisions about the type of treatment were taken according to local practices and there were no standard regional protocols. The choice of CABG technique, performed either with the use of extracorporeal circulation (ECC) or off-pump, was left to the surgeon's discretion. Whenever possible, the left internal thoracic artery (LIMA) was used preferentially for revascularization of the left anterior descending artery (LAD). Complete revascularization was performed with other arterial conduits, namely, right internal mammary artery (RIMA) and radial artery (RA) or saphenous vein grafts.

Follow-up angiography was not performed routinely in either group of patients.

### 2.4. Definition of the Outcomes

All-cause death included overall mortality occurring during the index hospital admission or thereafter. Cardiac death was defined as any death due to a cardiac cause (e.g., myocardial infarction (MI), low output failure, and fatal arrhythmia), and other types were procedure-related death and death of unknown cause. Acute MI was defined as any hospital admission occurring after the index procedure with a principal diagnosis of MI. This adverse event is defined according to the recent definition criteria by Moussa et al. [14]. Stroke included complications at the index admission and further hospital admissions with stroke as principal diagnosis. Overall rehospitalization was defined as any hospital admission after the index procedure due to a cardiac cause (e.g., MI, repeat PCI, repeat CABG, new pacemaker implantation, new occurrence of heart failure, or need for long-term hospital care). Repeat PCI was defined as any percutaneous coronary procedure during follow-up, treating a luminal stenosis in the same coronary vessel treated at the index procedure or treating other native vessel stenosis not previously revascularized.

### 2.5. Statistical Analysis

The study population was classified into 4 different age bands: less than 60 years, 60 to 69 years, 70 to 79 years, and 80 years or older, respectively. Demographic and clinical features of the patients were presented as counts and percentages and were compared between the four age classes, using the Chi-square test or Fisher's exact test when appropriate. For each class of age, cumulative risk curves of assessed outcomes (death for all causes, including perioperative deaths, cardiac death, myocardial infarction, stroke, repeat revascularization with PCI, and repeat hospitalization) were estimated at 5 years using the Kaplan-Meier method and were also compared by log-rank test.

Patients were then split into two groups, using a cut-off age of 60 years. Multivariate logistic regression analysis, with a binary dependent variable representing <60 years versus >60 years, was performed to estimate propensity score (PS) of treatment. Independent variables included demographics and the available clinical characteristics. Patients were matched on the logit of the PS using a caliper of width equal to 0.25 standard deviations of the logit of PS. Appropriateness of the specification of the PS was assessed by examining the degree to which matching on the estimated PS resulted in a matched sample in which the distribution of measured baseline covariates was similar between the two groups. Imbalances in baseline covariates were detected by standardized differences. Standardized differences of less than 0.10 (10%) are likely to indicate a negligible imbalance between the two groups.

Kaplan-Meier estimates were used to plot the rates of the long-term adverse events, and differences between risk curves were assessed using the Klein-Moeschberger test for matched pairs. Independent predictors of 5-year mortality risk were estimated using a stepwise multivariable Cox proportional hazards model, with robust standard errors to account for clustering in matched pairs including treatment and individual covariates including all pre- and intraoperative variables available.

All the analyses were performed with SAS version 9.3.

## 3. Results

**Figure 2:**
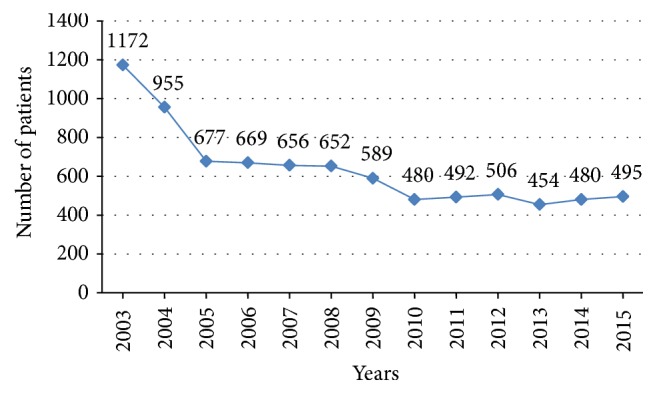
CABG trend over years in Emilia-Romagna region (Italy).

A reduction in the number of overall isolated elective CABG was observed in our registry between 2003 and 2015, although a plateau can be seen in the last five years (Figure 2).

The entire study cohort showed that patient risk profiles differed significantly between the groups (Table 1). The prevalence of patients under 60 is 18.9% (1564 of 8277 patients). Patients over 60 show a significantly lower prevalence of baseline comorbidities (Table 1). In particular, female sex, chronic pulmonary disease, extracardiac arteriopathy, and neurologic dysfunction disease were significantly less frequent in this younger population (Table 1). On the other hand, the prevalence of BMI ≥ 30 Kg/m^2^ was significantly higher in the under 60 group. Previous MI was reported in about one-third of patients < 60 and was significantly higher than in patients aged 60–69 and 70–79 years. Moreover, patients < 60 reported more frequently a preoperative severe depressed left ventricular ejection fraction (LVEF), although this was not statistically significant. They also reported a significantly more frequent history of previous PCI. However, older patients presented at surgery more often with higher EuroSCORE, due to the higher incidence of systemic comorbidities. We found that coronary revascularization was performed off-pump more frequently in patients over 60, whereas patients under 60 received more frequently on-pump total arterial revascularization (Table 1).

At 30 days, mortality was 0.4% in patients under 60 (6 patients), 0.7% in those aged 60 to 69 years (20 patients), 2% in those aged 70 to 79 years (64 patients), and 3% in those aged 80 years or older (18 patients).

The mean follow-up of the overall cohort was 8.1 ± 3.9 years (median 8.26, min 1.2–max 14.4).

**Figure 3:**
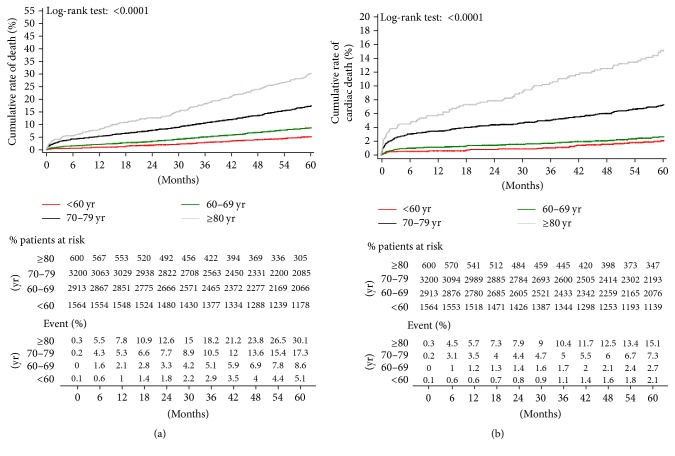
Kaplan-Meier estimates of late outcome in overall study population. (a) cumulative all-cause death; (b) cardiac-related death.

**Figure 4:**
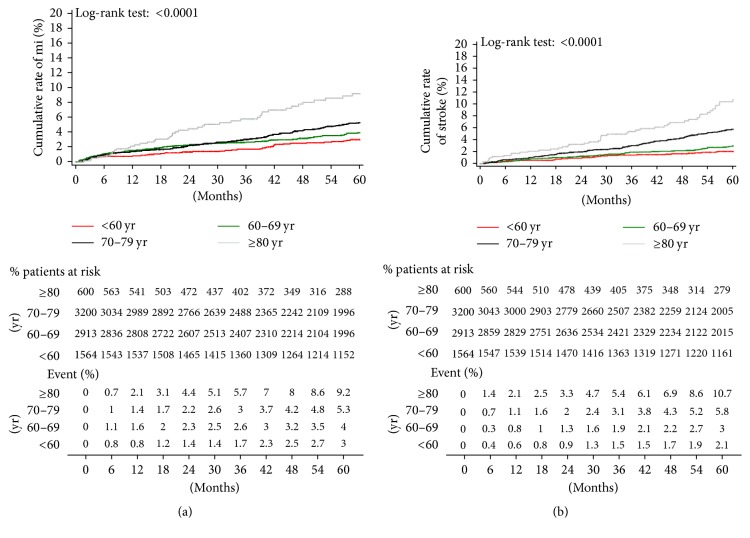
Kaplan-Meier estimates of late outcome in overall study population. (a) Myocardial infarction; (b) stroke.

**Figure 5:**
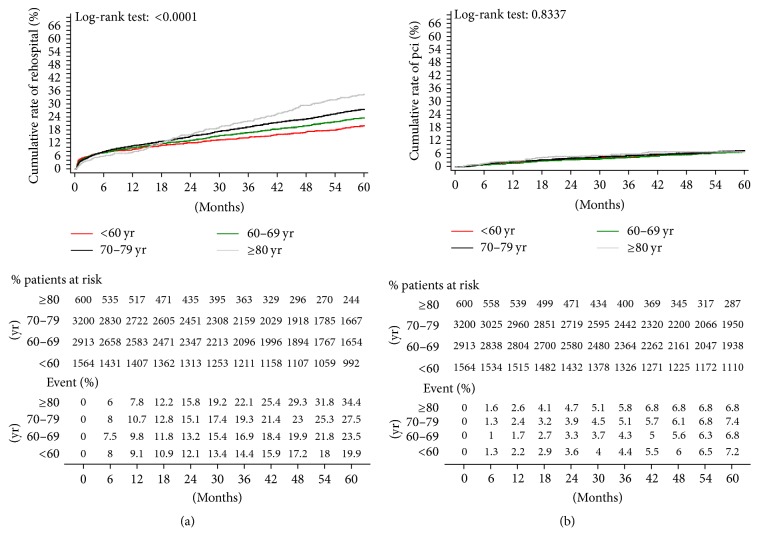
Kaplan-Meier estimates of late outcome in overall study population. (a) Repeat hospitalization; (b) repeat revascularization with PCI.

The unadjusted estimates of 5-year outcomes are summarized in Figures 3 and 4. Younger patients reported significant better results than older patients in terms of overall mortality (Figure 3(a)), cardiac-related mortality (Figure 3(b)), AMI (Figure 4(a)), stroke (Figure 4(b)), and rehospitalization (Figure 5(a)). However, no difference between groups is detected in terms of need for repeat PCI (Figure 5(b)). Log-rank test *p* values were significant for all outcomes considered except repeat PCI, confirming the heterogeneity of outcome curves between age groups.

As a second step we performed 1 : 1 propensity score matching for patients under 60 and patients over 60, in order to compare the outcomes of both groups with similar baseline and operative characteristics, except age. This matching yielded a cohort of 3128 patients, 1564 for each group (Table 2).

**Figure 6:**
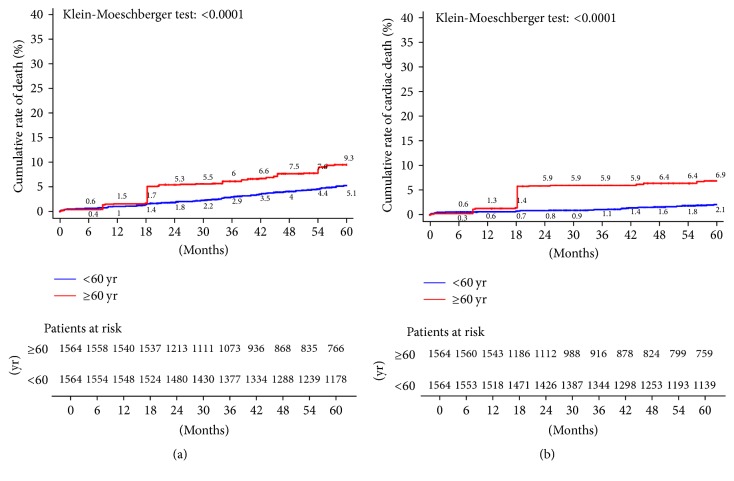
Kaplan-Meier estimates of late outcome in propensity score-matched pairs. (a) Cumulative all-cause death; (b) cardiac-related death.

**Figure 7:**
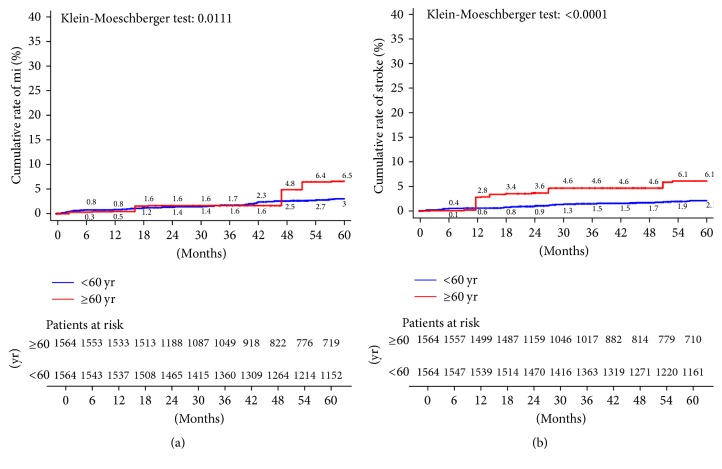
Kaplan-Meier estimates of late outcome in propensity score-matched pairs. (a) myocardial infarction; (b) stroke.

**Figure 8:**
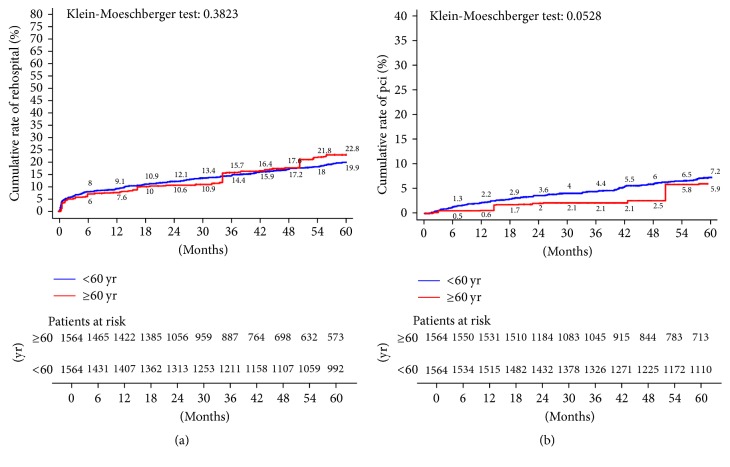
Kaplan-Meier estimates of late outcome in propensity score-matched pairs. (a) Repeat hospitalization; (b) repeat revascularization with PCI.

In this PS matched population, patients under 60 reported significantly lower overall mortality (at 5 years, 5.1% versus 9.3%; KM log-rank test *p* < 0.0001) (Figure 6(a)), cardiac-related mortality (at 5 years, 2.1% versus 6.9%; KM log-rank test *p* < 0.0001) (Figure 6(b)), incidence of AMI (at 5 years, 3% versus 6.5%; KM log-rank test *p* = 0.01) (Figure 7(a)), and stroke rates (at 5 years, 2.1% versus 6.1%; KM log-rank test *p* < 0.0001) (Figure 7(b)). No differences were reported between groups in terms of need for rehospitalization (at 5 years, 19.9% versus 22.8%; KM log-rank test *p* = 0.38) (Figure 8(a)). Patients under 60 required repeated revascularization more frequently than older patients (at 5 years, 7.2% versus 5.9%; KM log-rank test *p* = 0.05) (Figure 8(b)).

Table 3 reports multivariate analysis with significant independent predictors of mortality at 5 years.

## 4. Discussion

Recent studies conducted in Western Countries have found that the incidence of CAD has declined in the general population over the last few decades [15, 16], probably due to better prevention of cardiovascular risk. On the other hand, the incidence of CAD, including acute coronary artery syndromes, among young to middle-aged adults has been shown to have increased [15–17]. Previous studies of CAD in young adults have mostly been single-center analyses [3, 6], and few have been designed with the aim of studying young patients undergoing coronary revascularization [3, 5, 7, 18–20].

The aim of this retrospective multicenter registry study was to investigate age-dependent trends in mortality, long-term survival, and comorbidity over time in a large population of patients undergoing isolated CABG.

The main findings of this study are as follows. Patients under 60 years of age who underwent CABG had lower long-term mortality and morbidity than older patients. Of particular interest is that at 5 years the <60 group reported unadjusted significantly lower cumulative rates of all-cause death, cardiac-related death, AMI, stroke, and rehospitalization. No difference between groups was detected in terms of need for repeat PCI.

In the matched population, patients under 60 reported significantly lower overall mortality, lower cardiac-related mortality, lower incidence of AMI, and a lower stroke rate. However, no differences were reported between groups in terms of need for rehospitalization. Finally, patients under 60 required repeated revascularization more frequently than older patients.

It is well known that cardiovascular risk factors vary with regard to their impact on age of presentation with CAD. Our study confirms that patients younger than 60 have a different clinical pattern of presentation of CAD in comparison with elderly patients. Particularly, male gender, obesity, the history of previous myocardial infarction, the presence of depressed LVEF, and a history of previous PCI have been found to be highly prevalent among patients < 60, confirming the results of a recent study by Moussa et al. [14].

Obesity has already been recognized as an independent risk factor for CAD [21], and recently a close association between severity of obesity, measured by BMI > 30 kg/m^2^, and a progressive reduction in the mean age of patients with symptomatic CAD has been demonstrated [22]. In particular, abdominal obesity has been found to be closely associated with the risk of myocardial infarction [23] and this is observed more often in men [24]. On the basis of these observations and our results, it is reasonable to postulate that the risk of CAD due to obesity may be higher in men than women. The higher proportion of males in the young population undergoing CABG in our study is not surprising, given that it is widely recognized that CAD occurs 7 to 10 years earlier in men than women [25, 26].

On the other hand, systemic comorbidities usually associated with severe CAD, such as chronic pulmonary disease, diabetes, stroke, and extracardiac arteriopathy in our study, proved to be less frequent in patients aged <60. This is consistent with the previous international literature [27, 28] and may be explained by the fact that the onset of diabetes mellitus and systemic hypertension usually occurs later in life, and their effect on the pathogenesis of CAD may require several years or decades to become clinically evident. The purpose of this study was not to primarily investigate the risk factors determining premature coronary artery disease, but our findings clearly confirm that the pathogenesis of coronary artery disease remains complex and that both genetic and environmental factors contribute to the early onset of coronary artery disease.

Long-term mortality was considerably lower in patients < 60 years than in patients > 60, and this result is consistent with mortality rates reported in previous studies of young patients undergoing CABG [7, 14, 17, 29]. The long-term efficacy of CABG in patients < 60 is further confirmed by a significantly higher freedom from AMI and stroke in comparison to older patients. Systemic comorbidities usually associated with severe CAD, such as chronic pulmonary disease, diabetes, and extracardiac arteriopathy in our study, were found to be less frequent in patients aged <60, and this contributes to the lower postoperative and long-term morbidity reported in this subgroup of patients. In fact, in our study multivariate analysis confirmed that all classic clinical cardiac conditions and systemic comorbidities (severely depressed left ventricular function, history of previous myocardial infarction, NYHA classes III-IV, chronic renal failure, diabetes, chronic pulmonary disease, extra-cardiac arteriopathy, previous CABG, revascularization with off-pump technique, and left main coronary disease) are independent risk factors for mortality at 5 years.

Only 27.4% of patients < 60 years received complete arterial graft revascularization in our study. A large body of scientific evidence demonstrates survival and repeat revascularization benefits of bilateral internal mammary artery grafting [30, 31]. It is however the case that results of a recent ART Trial did not confirm significantly better mortality and morbidity rates at 5 years in patients who received bilateral mammary artery grafts in comparison to those, with similar mean age, who had traditional revascularization with single mammary artery grafts and saphenous vein grafts [32]. Nevertheless, in our study patients under 60 received total arterial revascularization more frequently than other age subgroups, and this may have potentially contributed to better long-term outcomes reported in this subgroup of patients. However, it appears that the clearest survival benefit of bilateral internal mammary artery grafting occurs after 5 years of follow-up and continues to increase 20 years after CABG [31].

The significantly higher rate of total arterial revascularization, which does not usually require intraoperative aortic manipulation, and the lower rate of extracardiac arteriopathy reported in younger patient subgroups may also partly explain the significantly better stroke rates in patients under 60.

Despite the favorable outcomes reported in patients < 60 undergoing CABG, this study demonstrated that repeated revascularization occurred similarly in all the subgroups of unadjusted population and was more frequent in patients < 60 after PS matching. Possible explanations involve the lower amount of daily activity of older patients, so that they are less exposed to the potential recurrence of angina and the reluctance of older patients to undergo repeat revascularization procedures. On the other hand, younger patients are generally followed more closely by the cardiologist, with more frequent check-ups, and they may be given more aggressive medical therapy, but it is also reasonable to admit that they have a lower threshold for performing repeat revascularization in the case of mild angina symptoms.

The strength of this study is that it is a regional multicenter study including almost all consecutive patients undergoing isolated CABG surgery in Emilia-Romagna region (Italy) in a 12-year period. The study population is large, and there is a long follow-up period (mean follow-up 8.1 years).

There are, however, several limitations inherent in the observational design of the study which need to be acknowledged.

We had no information about the patients not resident in the ER region and excluded from the follow-up analysis because of incomplete information on their clinical status after discharge from hospital. Although we tried to rigorously adjust selection bias using propensity score-based analysis, unmeasured confounders and hidden biases may have affected our results. The choice of strategy to perform CABG (off- versus on-pump CABG; total arterial revascularization versus traditional revascularization) was strongly affected by the surgeons' preferences, as well as by several other important baseline demographic and clinical profiles of the patients enrolled in the entire study cohort. The propensity score analysis could thus be slightly imperfect, and we were unable to completely adjust for hidden selection biases. Moreover, only clinical outcomes were assessed in this study, and graft patency was not assessed during the follow-up. Finally, we have no information on vessels (grafts or native coronary arteries) requiring repeat revascularization in the follow-up.

However, it should be recognized that some end-points as cardiac-related death and nSTEMI are not so easy to define and to collect in the follow-up of such a retrospective study, constituting a limitation in the evaluation of adverse outcomes in this patient population.

In conclusion, patients < 60 years of age who underwent CABG had a lower risk of adverse outcomes than older patients. Of particular interest is that at 5 years the <60 group reported unadjusted and adjusted significantly lower cumulative rates of all-cause death in comparison to other groups. Patients < 60 have a different clinical pattern of presentation of CAD in comparison with elderly patients. In particular, male gender, obesity, the history of previous myocardial infarction, the presence of depressed left ventricular function, and the history of previous PCI were found to be very prevalent among patients < 60. These issues require great attention in the design and improvement of preventive strategies for reducing the impact of specific cardiovascular risk factors for younger patients, such as diet, lifestyle, and weight control.

Further studies with longer follow-up periods need to be conducted with the aim of studying the efficacy and durability of myocardial revascularization in younger patients who require CABG.

## Figures and Tables

**Table 1:** Baseline characteristics and operative data of patients according to different age classes.

Patients' characteristics	<60 yrs		60–69 yrs		70–79 yrs		≥80 yrs		*p*
	(*N* = 1564)		(*N* = 2913)		(*N* = 3200)		(*N* = 600)		
	*N*	%	*N*	%	*N*	%	*N*	%	
Female	154	*9.8*	416	*14.3*	726	*22.7*	181	*30.2*	*<0.0001*
BMI ≥ 30 kg/m^2^: obesity	402	*25.7*	595	*20.4*	486	*15.2*	58	*9.7*	*<0.0001*
Logistic EUROscore > 15%	3	*0.2*	36	*1.2*	205	*6.4*	136	*22.7*	*<0.0001*
Critical preoperative state	21	*1.3*	30	*1.0*	45	*1.4*	10	*1.7*	*0.457*
Unstable angina	101	*6.5*	203	*7.0*	243	*7.6*	85	*14.2*	*<0.0001*
LVEF ≤ 30%	44	*2.8*	75	*2.6*	73	*2.3*	13	*2.2*	*0.661*
LVEF 30%–60%	370	*23.7*	697	*23.9*	869	*27.2*	209	*34.8*	*<0.0001*
Previous myocardial infarction	464	*29.7*	695	*23.9*	877	*27.4*	188	*31.3*	*<0.0001*
Serum creatinine ≥ 2 mg/dl	46	*2.9*	105	*3.6*	120	*3.8*	22	*3.7*	*0.548*
Diabetes	389	*24.9*	854	*29.3*	898	*28.1*	128	*21.3*	*<0.0001*
Systolic PA pressure > 60 mmHg	13	*0.8*	30	*1.0*	38	*1.2*	2	*0.3*	*0.231*
Chronic pulmonary disease	47	*3.0*	124	*4.3*	221	*6.9*	38	*6.3*	*<0.0001*
NYHA III-IV	144	*9.2*	267	*9.2*	363	*11.3*	81	*13.5*	*0.001*
Extracardiac arteriopathy	229	*14.6*	697	*23.9*	1013	*31.7*	191	*31.8*	*<0.0001*
Neurological dysfunction disease	80	*5.1*	182	*6.2*	197	*6.2*	50	*8.3*	*0.048*
Previous cardiac surgery	19	*1.2*	52	*1.8*	79	*2.5*	10	*1.7*	*0.022*
Single-vessel disease	159	*10.2*	281	*9.6*	347	*10.8*	93	*15.5*	*0.000*
Double-vessel disease	644	*41.2*	1141	*39.2*	1273	*39.8*	235	*39.2*	*0.610*
Triple-vessel disease	761	*48.7*	1491	*51.2*	1580	*49.4*	272	*45.3*	*0.048*
Previous PCI	280	*17.9*	427	*14.7*	463	*14.5*	73	*12.2*	*0.002*
Previous CABG	14	*0.9*	40	*1.4*	65	*2.0*	7	*1.2*	*0.014*
Previous valve surgery	2	*0.1*	2	*0.1*	6	*0.2*	1	*0.2*	*0.642*
Off-pump	95	*6.1*	145	*5.0*	285	*8.9*	72	*12.0*	*<0.0001*
LMCA disease	31	*2.0*	80	*2.7*	104	*3.3*	28	*4.7*	*0.005*
Complete arterial grafts revascularization	428	*27.4*	643	*22.1*	524	*16.4*	96	*16.0*	*<0.0001*

BMI: body mass index; LVEF: left ventricular ejection fraction; PA: pulmonary artery; NYHA: New York Heath Association; PCI: percutaneous coronary intervention; CABG: coronary artery bypass grafting; LMCA: left main coronary artery.

**Table 2:** Baseline characteristics and operative data of patients as adjusted by multiple propensity score.

Patients' characteristics	<60 (*N* = 1564)		≥60 (*N* = 1564)		*p* value	Standardized differences
Female	154	*9.8*	161	*10.3*	0.6775	−0.015
BMI ≥ 30: obesity	402	*25.7*	409	*26.2*	0.7752	−0.01
Logistic EUROscore > 15%	3	*0.2*	0	*0.0*	0.0831	0.062
Critical preoperative state	21	*1.3*	12	*0.8*	0.1152	0.056
Unstable angina	101	*6.5*	76	*4.9*	0.053	0.069
Ejec. fraction ≤ 30%	44	*2.8*	35	*2.2*	0.3051	0.037
Ejec. fraction 30%–60%	370	*23.7*	375	*24.0*	0.8338	−0.008
Previous myocardial infarction	464	*29.7*	470	*30.1*	0.8147	−0.008
Serum creatinine ≥ 2 mg/dl	46	*2.9*	15	*1.0*	<0.0001	0.144
Diabetes	389	*24.9*	390	*24.9*	0.967	−0.001
Systolic PA pressure > 60 mmHg	13	*0.8*	4	*0.3*	0.0286	0.078
Chronic pulmonary disease	47	*3.0*	100	*6.4*	<0.0001	−0.161
NYHA III-IV	144	*9.2*	148	*9.5*	0.8058	−0.009
Extracardiac arteriopathy	229	*14.6*	273	*17.5*	0.0321	−0.077
Neurological dysfunction disease	80	*5.1*	391	*25.0*	<0.0001	−0.579
Previous cardiac surgery	19	*1.2*	8	*0.5*	0.0335	0.076
Single-vessel disease	159	*10.2*	94	*6.0*	<0.0001	0.153
Double-vessel disease	644	*41.2*	790	*50.5*	<0.0001	−0.188
Triple-vessel disease	761	*48.7*	680	*43.5*	0.0037	0.104
Previous PCI	280	*17.9*	277	*17.7*	0.8885	0.005
Previous CABG	14	*0.9*	5	*0.3*	0.0384	0.074
Previous valve intervention	2	*0.1*	1	*0.1*	0.5635	0.021
Off-pump	95	*6.1*	87	*5.6*	0.5412	0.022
LMCA disease	31	*2.0*	27	*1.7*	0.596	0.019
Total arterial revascularization	428	*27.4*	430	*27.5*	0.9361	−0.003

BMI: body mass index; LVEF: left ventricular ejection fraction; PA: pulmonary artery; NYHA: New York Heath Association; PCI: percutaneous coronary intervention; CABG: coronary artery bypass grafting; LMCA: left main coronary artery.

**Table 3:** Predictors for 5-years mortality risk (Cox proportional hazards model).

Parameter	Hazard ratio	95% hazard ratio confidence interval		*p*
Age < 60 yrs	0.3	0.2	0.5	<0.0001
LVEF ≤ 30%	2.2	1.4	3.6	0.002
Previous myocardial infarction	1.3	1.1	1.6	0.004
Serum creatinine ≥ 2 mg/dl	2.2	1.5	3.2	<0.0001
Diabetes	1.5	1.3	1.8	<0.0001
Chronic pulmonary disease	1.8	1.3	2.5	0.0002
NYHA III-IV	1.5	1.2	2.0	0.001
Extracardiac arteriopathy	1.7	1.4	2.1	<0.0001
Previous CABG	2.5	1.3	4.8	0.0069
Off-pump	2.3	1.6	3.4	<0.0001
LMCA disease	2.3	1.2	4.4	0.013

LVEF: left ventricular ejection fraction; NYHA: New York Heath Association; CABG: coronary artery bypass grafting; LMCA: left main coronary artery.
